# Attachment styles, grief responses, and the moderating role of coping strategies in parents bereaved by the Sewol ferry accident

**DOI:** 10.1080/20008198.2018.1424446

**Published:** 2018-01-19

**Authors:** Hyu Jung Huh, Kyung Hee Kim, Hee-Kyung Lee, Jeong-Ho Chae

**Affiliations:** ^a^ Department of Psychiatry, The Catholic University of Korea, College of Medicine, Seoul, Republic of Korea; ^b^ Department of Psychology, The Catholic University of Korea, Bucheon, Republic of Korea

**Keywords:** Traumatic loss, attachment, coping strategy, complicated grief, disaster, Perdida traumática, vinculo, estrategia de afrontamiento, duelo complicado, Desastre, 创伤丧失, 依恋, 应对策略, 复杂哀伤, 灾难, • Participants in both the EMDR and CBT groups reported positive changes in their experience of grief and quality of life following treatment.• Some of the changes described were unique to each treatment condition, and these aligned with the underlying theories of EMDR and CBT respectively.• CBT participants reported acquiring tools and skills to regulate emotions.• EMDR participants reported an increased distancing from their upsetting memories.

## Abstract

**Background**: Previous studies on the influence of different types of attachment on grief responses have yielded contradictory outcomes. Little research has been conducted to identify the psychological processes that moderate the relationship between attachment representations and patterns of grief in disaster-related grief.

**Objective**: The present study examines the effects of different attachment types on the grief responses of parents bereaved by loss of a child in a ferry accident, along with the moderating role of coping strategies.

**Methods**: Bereaved parents (*n* = 81) completed self-report questionnaires evaluating attachment, coping strategies, complicated grief, and shame/guilt. We performed correlational analyses to examine the associations among variables. We also conducted hierarchical regression analyses and simple slope analyses to examine the moderation effects of coping strategies.

**Results**: Anxious attachment was associated with severe shame/guilt, and avoidant attachment correlated with complicated grief. Anxious attachment was positively associated with all types of coping strategies, and avoidant attachment was negatively related to problem- and emotion-focused coping. The use of problem-focused coping strategies was a significant moderator of the relationship between the avoidant attachment dimension and shame/guilt. Avoidant attachment had a significant effect on shame/guilt in groups with a high level of problem-focused coping. In contrast, none of the coping strategies significantly moderated the relationship between anxious attachment and grief response.

**Conclusions**: The results suggest that people with highly avoidant attachment might be overwhelmed by shame and guilt when they try to use problem-focused coping strategies. This finding suggests that grief interventions should be organized with consideration of individual differences in attachment representations.

## Abstract (zh)

**Contexto**: estudios previos realizados sobre la influencia de diferentes tipos de vinculo en el marco de respuestas de duelo han dado resultados contradictorios. Se han realizado hasta ahora escasas investigaciones para identificar los procesos psicológicos que moderan la relación entre representaciones vinculares y patrones de duelo en los casos de duelo relacionado con un suceso catastrófico.

**Objetivo**: El presente estudio examina los efectos de diferentes tipos de vinculación sobre las respuestas de duelo de padres afligidos por la perdida de un hijo en un accidente de un ferry. También se estudia el papel moderador de estrategias de afrontamiento.

**Métodos**: Los padres en duelo (*n* = 81) completaron varios cuestionarios auto administrados que evalúan vinculo, estrategias de afrontamiento, duelo complicado así como vergüenza/culpa.

**Resultados**: La vinculación de tipo ansioso se asoció con sentimientos severos de vergüenza/culpa. Por otro lado, el tipo de vinculación evitativa se correlacionó con duelo complicado. El vinculo de tipo ansioso se asoció positivamente con todo tipo de estrategias de afrontamiento. La vinculación evitativa se relacionaba negativamente con el afrontamiento focalizado en el problema y en la emoción. El uso de estrategias de afrontamiento focalizadas en el problema fue un moderador significativo de la relación entre la dimensión vinculo evitativo y vergüenza/culpa. El vinculo evitativo tuvo un efecto significativo sobre la vergüenza/culpa en grupos con un nivel alto de coping focalizado en el problema. Por contraste, ninguna de las estrategias de afrontamiento moderaban de forma significativa la relación entre vinculo ansioso y respuesta de duelo.

**Conclusiones**: Los resultados sugieren que las personas con un tipo de vinculación altamente evitativa pueden sentirse superados por la vergüenza cuando intentan usar estrategias de afrontamiento focalizadas en el problema. Este hallazgo sugiere que las intervenciones en el duelo deben ser organizadas teniendo en cuenta las diferencias individuales en las representaciones de vinculo.

## Abstract (es)

背景：关于不同依恋类型对哀伤反应的影响的前人研究揭示出矛盾的结果。很少有研究关注哪些心理过程可以调节依恋表现和与灾难相关的哀伤模式之间的关系。

目标：本研究考察了在一次渡轮事故中失去一个孩子的父母样本中，不同依恋类型对哀伤反应的影响，和应对策略的调节作用。

方法：81名丧子父母完成了评估依恋、应对策略、复杂哀伤和羞耻/罪疚的自评问卷。我们进行了回归分析考察变量间的关联性。我们还进行了层级回归分析和简单斜率分析来考察应对策略的调节作用。

结果：焦虑依恋和严重的羞耻/罪疚相关，回避依恋和复杂哀伤相关。焦虑依恋和所有的应对策略类型正相关，回避依恋和问题中心以及情绪中心应对方式负相关。使用问题中心应对策略是回避依恋问题和羞耻/罪疚的显著调节变量。回避依恋对使用高水平问题中心应对者的羞耻/罪疚有显著影响。相反的，任何一个应对策略都没有显著调节焦虑依恋和哀伤反应之间的关系。

结论：结果显示高回避依恋者在试图使用问题中心应对策略时会被羞耻和罪疚感压倒。这个发现提示哀伤干预应该考虑依恋表现的个体差异。

## 1. Introduction

A 6825-ton motor vessel, the Sewol ferry, sank in the sea of Jeollanam-do, South Korea, on 16 April 2014. Of the 476 passengers and crewmembers onboard, 304 died in the disaster. Of the total passengers, 339 were high school students and teachers on a field trip, 261 of whom died in the accident. More than 500 parents lost a child in the accident, and they have suffered serious complicated grief problems as a result of the traumatic loss (Huh, Huh, Lee, & Chae, 2017). The disaster provoked various negative, complicated emotions in parents, including anger, guilt, and shame. As the ship capsized, the ferry made an announcement ordering the passengers to stay put, warning that moving was dangerous, and that announcement continued to be broadcast even as the ship began flooding. Parents who told their children by phone to obey the announcements experienced excessive guilt after the accident. After the accident, the bereaved families protested as a group and called for the establishment of an independent committee to investigate the government’s responsibility for the disaster. Their long-term collective actions might have contributed to their social identity and stigma. Furthermore, the substantial amount of media attention and related misunderstandings or criticisms about such parents might have contributed to their shame.

The sudden death of a loved one through such a human-caused accident can complicate the grief process compared to a loss after an illness (Boelen, de Keijser, & Smid, 2015; Jacobs, Mazure, & Prigerson, 2000; Neria et al., 2007; Prigerson et al., 1997). Furthermore, the sudden loss of a child in an accident is often extremely difficult because children are often closely related to a parent’s sense of self and identity, including how they views themselves in the past, present, and future (Boelen, 2016). In this context, *self-conscious emotions*, feelings defined in a self-evaluative process in relation to societal standards for behaviour, might be important in the grieving process of bereaved parents. Previous studies have reported that negative self-conscious emotions, including shame and guilt, are commonly experienced by bereaved parents and disturb the normal progression of grieving (Barr, 2004, 2012). However, few empirical studies have considered the relationships among complicated grief, negative self-conscious emotions, and related individual factors in bereaved parents whose traumatic loss was caused by a disaster.

Among the several factors that influence individual differences in grief reactions and emotions, previous research shows that attachment style is important (Wijngaards-de Meij et al., 2007a). Attachment theory suggests that individual attachment representations influence the processes, patterns, and outcomes of emotion regulation while grieving (Bowlby, 1980). Attachment models have been conceptualized with two dimensions that underlie adult attachment styles: avoidant attachment and anxious attachment (Bowlby, 1980). Individuals with a highly avoidant attachment style generally struggle for independence and maintain emotional distance from significant others (Bartholomew, 1990; Mikulincer, Birnbaum, Woddis, & Nachmias, 2000; Mikulincer, Gillath, & Shaver, 2002). Individuals with a highly anxious attachment style are excessively dependent on significant others and worry that persons in close relationships with them will not be available or supportive in stressful times (Fraley & Shaver, 1997; Mikulincer et al., 2000).

Several empirical studies have been performed on this topic. Anxious attachment has been found to be related to poorer outcomes in bereavement (Field & Sundin, 2001; Fraley & Bonanno, 2004; Wayment & Vierthaler, 2002). However, previous findings about avoidant attachment have been inconsistent. In one study, individuals with avoidant-attachment displayed fewer grief, depression, anxiety, and posttraumatic symptoms than individuals with anxious attachment. In other studies, the avoidant attachment style was associated with prolonged grief (Boelen & Klugkist, 2011; Jerga, Shaver, & Wilkinson, 2011).

Many researchers have considered coping strategies as an important factor in the bereavement process. According to the theoretical background, one coping style that may be important in adjustment to bereavement is avoidance. A few studies provide evidence of a relationship between the avoidant coping style and grief-related responses (Boelen, van den Bout, & van den Hout, 2003; Schnider, Elhai, & Gray, 2007). Several studies have also examined the relationships among attachment style, coping strategies, and grief responses (Boelen, van den Bout, & van den Hout, 2006, 2010). One study reported that several cognitive processes related to avoidant coping strategies mediated the effect of avoidant attachment on grief response (van der Houwen, Stroebe, Schut, Stroebe, & van den Bout, 2010).

According to stress-coping theory, coping processes include two types of strategies: problem-focused and emotion-focused coping (Lazarus, 1993). Problem-focused coping is active and involves solving problems. Emotion-focused coping has two types: active and avoidant (Holahan & Moos, 1987). Active emotional coping involves expressing one’s emotions or cognitively reframing a stressful situation. Avoidant emotional coping involves suppressing or avoiding emotions. Persons with anxious attachment might have a tendency to use active emotional coping and some problem-focused coping strategies because they are hyper-vigilant, repetitively thinking about negative events, exaggerating painful emotions, and depending on others for support (Suar, Alat, & Das, 2017). Individuals with avoidant attachment might use distraction or disengagement strategies, such as distancing themselves from and suppressing their emotions and memories, making them more likely to use avoidant coping strategies (Suar et al., 2017).

In the present study, we investigated the relationships between the two dimensions of attachment and grief response in the parents bereaved by the Sewol ferry accident. We also examined the moderating effects of coping strategies on the relationship between attachment and complicated grief and shame/guilt. Our hypotheses were: (1) both types of insecure attachment will be positively associated with the severity of complicated grief and shame/guilt, and (2) the moderating effects of coping strategy will differ according to attachment style. Theoretical and empirical studies suggest that avoidant strategies, often preferred by avoidant-attached persons, can be related to negative grief outcomes. Therefore, we expected avoidant strategies to strengthen the association between avoidant attachment and complicated grief, shame, and guilt. In contrast, we expected the adaptive/active form of coping to promote good grief outcomes, with anxious attachment more associated with those forms of coping than avoidant attachment. Therefore, we expected that the active/adaptive forms of problem- and emotion-focused coping strategies would weaken the association between insecure attachment and grief response, especially between anxious attachment and grief response.

## 2. Methods

### 2.1. Participants and procedures

The present investigation was performed as part of the disaster cohort study of the mental health problems of persons affected by disasters, supported by the Korean Mental Health Technology R&D Project, Ministry of Health & Welfare. Before the study, the researchers contacted representatives of the bereaved families to explain the purpose of the study and obtain their agreement to participate. Then, study recruitment, survey scheduling, and questionnaire dissemination were conducted with the cooperation of the Ansan Mental Health Trauma Center, established to provide long-term mental health care for bereaved families. A total of 81 bereaved parents who agreed to participate and completed all of the questionnaires were included in the study.

The survey was performed at a mean of 18 months (*SD* = 1 month) after the accident. Before the survey, the interviewers obtained informed consent from all participants, including an explanation of the aim and importance of the study. The study procedure was approved by the Institutional Review Board of the Ethical Committee at Seoul St. Mary’s Hospital at The Catholic University of Korea.

### 2.2. Measures

We surveyed the participants regarding demographic variables (age, gender, years of education, marital status, employment status). We also evaluated the different styles of attachment, coping strategies, and trauma-related emotions using the measures described below.

### 2.3. Attachment

We assessed the severity of the different styles of insecure attachment using the Experiences in Close Relationships Questionnaire-Short Form (ECR-SF) (Wei, Russell, Mallinckrodt, & Vogel, 2007). The ECR-SF is a 12-item measure that assesses adult attachment using a 7-point Likert scale ranging from 1 (*strongly disagree*) to 7 (*strongly agree*). Participants were instructed to respond considering how they generally experienced their relationships with their deceased children. Items were grouped into two scales: attachment anxiety and attachment avoidance. In the present study, the Cronbach’s alphas for the attachment anxiety and avoidance scales were 0.678 and 0.697, respectively.

### 2.4. Coping strategy

To evaluate responses coping with stress after the accident, we used the Brief COPE (Carver, 1997), a self-report questionnaire used to assess the different coping behaviours and thoughts a person could have in response to a specific situation. It is made up of 14 subscales: self-distraction, active coping, denial, substance use, use of emotional support, use of instrumental support, behavioural disengagement, venting, positive reframing, planning, humour, acceptance, religion, and self-blame. Participants rated 28 coping behaviours and thoughts (two items for each subscale) on frequency of use with a scale from 1 (*I haven’t been doing this at all*) to 4 (*I’ve been doing this a lot*). The wording was modified for the present study to fit the population and the challenges the participants faced. Based on the theoretical literature and several empirical studies, we grouped the 14 subscales into three categories: (1) problem-focused coping (active coping, planning, instrumental support), (2) emotion-focused coping (positive reframing, humour, religion, acceptance, emotional support), and (3) avoidant coping (self-blame, behavioural engagement, substance abuse, self-distraction, denial, venting) (Carver & Scheier, 1994; Carver, Scheier, & Weintraub, 1989; Coolidge, Segal, Hook, & Stewart, 2000; Cooper, Katona, & Livingston, 2008; Endler & Parker, 1994; Folkman & Lazarus, 1985; Schnider et al., 2007). In the present sample, the Cronbach alpha for problem-focused coping, emotion-focused coping, and avoidant coping was 0.863, 0.822, and 0.723, respectively.

### 2.5. Complicated grief

The Inventory of Complicated Grief (ICG) was used to evaluate severity of pathological grief (Prigerson et al., 1995). The instrument consists of 19 items about immediate bereavement-related thoughts and behaviours and presents five response options ranging from ‘never’ to ‘always’. In the present sample, the Cronbach alpha for the ICG was 0.925.

### 2.6. Shame and guilt

The Personal Feelings Questionnaire-2 (PFQ-2) was used to evaluate the trauma-related emotional responses of shame and guilt (Harder & Zalma, 1990). The PFQ-2, a revised version of the original PFQ, consists of two subscales: shame and guilt. Each item asks the subjects to respond on a 4-point Likert scale ranging from 0 (*you are not experiencing the feeling*) to 3 (*you are experiencing the feeling continuously or almost continuously*). In the PFQ-2, 10 items relate to the shame scale and six to the guilt scale. Although the PFQ-2 was developed to evaluate shame and guilt as different emotions, we investigated shame/guilt as negative self-conscious emotions that might play important roles in the bereavement process. It was not our intention to investigate on whether the effect of attachment and coping strategy differed between shame and guilt. Therefore, we used the PFQ-2 as a single index in the present study. For our sample, the Cronbach’s alpha for the PFQ-2 was 0.920.

### 2.7. Data analysis

**Figure 1.**
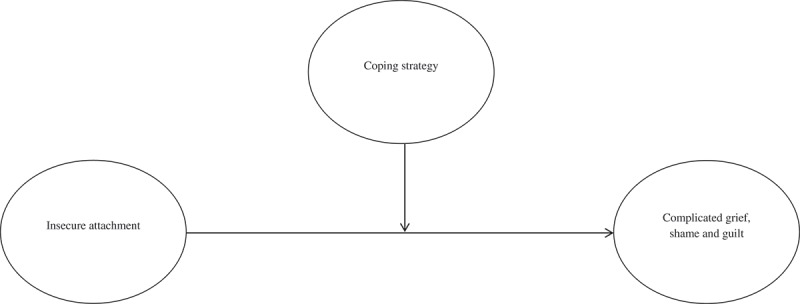
Hypothesized model about moderating role of coping strategy in the relationship between attachment and trauma related emotions.

Our main predictive variables were the levels of anxious and avoidant attachment. The moderating variable was coping strategy, and the dependent variables were complicated grief and shame/guilt (Figure 1).

To examine the characteristics of the participants, we obtained descriptive statistics via frequency analysis. To determine whether all data met the assumption of normal distribution, we examined the skewness and kurtosis of the main variables. We found that the absolute values of skewness and kurtosis were not over ± 2 and the *z*-scores were not over ± 3.29, which means that the data does not show substantial departure from normality (George & Mallery, 2010; Tabachnick & Fidell, 2007) (Table 1). We conducted simple correlational analyses to determine the degrees of relationship between the variables.

**Table 1.** Characteristics of participants.

Characteristic	Mean (*SD*) %	Skewness	SE_skewness_	Z_skewness_	Kurtosis	SE_kurtosis_	Z_kurtosis_
Age	47.96 (4.27)						
Gender (Male)	45.7%						
Educational year	14.05 (2.77)						
Employment status (Unemployment)	28.8%						
Marital status (Married/Cohabited)	57.1%						
Anxious type attachment (ECR-S Anxiety)	16.56 (5.80)	0.31	0.27	1.14	−0.13	0.53	−0.25
Avoidant type attachment (ECR-S Avoidant)	22.65 (4.72)	0.07	0.27	0.27	1.57	0.53	2.95
Problem-focused coping (Brief COPE Problem-focused coping)	12.59 (4.40)	0.48	0.27	1.79	−0.10	0.53	−0.19
Emotion-focused coping (Brief COPE Emotion-focused coping)	17.73 (4.97)	0.66	0.27	2.47	0.86	0.53	1.63
Avoidant coping (Brief COPE Avoidant coping)	25.40 (6.21)	0.05	0.27	0.17	0.61	0.53	1.15
Complicated grief (ICG)	52.55 (14.38)	−0.67	0.27	−2.49	−0.24	0.53	−0.45
Shame and guilt (PFQ-2)	58.49 (11.80)	−0.42	0.27	−1.55-	−0.54	0.53	−1.02

ECR-S = Experience in close relationship questionnaire-short form; Brief COPE = Brief coping orientation to problems experienced; ICG = Inventory of complicated grief; PFQ-2 = Personal feeling questionnaire-2 *p < .05, **p < .01

To test for a moderation effect, we performed hierarchical regression analysis, as suggested by Aiken and West (1991). Considering the possible problematic effect of multicollinearity when interaction terms are included in a regression model, we produced an interaction term by mean-centring the sum of the scores of the predictive variables (anxious attachment and avoidant attachment) and the moderating variable (coping strategies). In the first step, we entered the predictive and moderating variables into the model. In the next step, we simultaneously included the predictive variables, moderating variable, and interaction terms to examine their effects on the dependent variables, complicated grief and shame/guilt. To examine the pattern of the moderation effect, we performed simple slope analysis. All analyses were performed using SPSS 22.0 (SPSS Inc., Chicago, IL, USA).

## 3. Results

### 3.1. Participant characteristics

Participant demographics are presented in Table 1. The mean age of the participants was 47.96 (± 4.27) years, and 45.7% of participants were male. A total of 19.5% participants were separated or divorced, and their mean years of education were 14.05 (± 2.77). Of the total participants, 23.5% reported unemployment after the accident and 19% reported a past psychiatric history. As described in a previous, related, study, most of the bereaved parents still reported severe symptoms of complicated grief, depression, and posttraumatic stress, even though 18 months had passed since the accident (Huh et al., 2017). The mean ECR anxiety subscore was 16.56 (± 5.80), and the mean ECR avoidant subscore was 22.65 (± 4.72). The mean subscores of the Brief COPE problem-focused coping, emotional-focused coping, and avoidant-coping were 12.59 (± 4.40), 17.73 (± 4.97), and 25.40 (± 6.21), respectively. The mean score of the PFQ-2 was 58.49 (± 11.80), and the mean score of the ICG was 52.55 (± 14.38).

#### 3.1.1. Bivariate correlations between type of attachment, proactive coping strategy, and trauma-related emotions

The correlation matrix for all variables is provided in Table 2. Attachment anxiety was positively correlated with shame/guilt (*r* = 0.32, *p* < .01), use of problem-focused coping (*r* = 0.25, *p* < .05), emotion-focused coping (*r* = 0.30, *p* < .01), and avoidant coping (*r* = 0.24, *p* < .05). Attachment avoidance was positively correlated with complicated grief (*r* = 0.26, *p* < .05) and negatively correlated with use of problem-focused coping (*r* = −0.25, *p* < .05) and emotion-focused coping (*r* = −0.22, *p* < .05). No significant correlation was observed between use of problem- or emotion-focused coping strategies and grief response (shame/guilt and complicated grief). The use of avoidant coping strategies was positively correlated with shame/guilt (*r* = 0.52, *p* < .01) and complicated grief (*r* = 0.34, *p* < .01).

**Table 2.** Correlations among different attachment types, coping strategies, complicated grief, and shame/guilt.

	Anxious attachment	Avoidant attachment	Problem-focused coping	Emotion-focused coping	Avoidant coping	Complicated grief	Shame and guilt
1. Anxious attachment	-						
2. Avoidant attachment	.19	-					
3. Problem-focused coping	.25*	−.25*	-				
4. Emotion-focused coping	.30**	−.22*	.73**	-		-	
5. Avoidant coping	.24*	.13	.25*	.29**	-		
6. Complicated grief	.11	.26*	−.06	−.09	.34**	-	
7. Shame and Guilt	.32**	.22	.05	.07	.52**	.43**	-

*p < .05, **p < .01

#### 3.1.2. Moderation effect of coping strategy on the relationships between type of attachment, complicated grief, and shame/guilt

**Figure 2.**
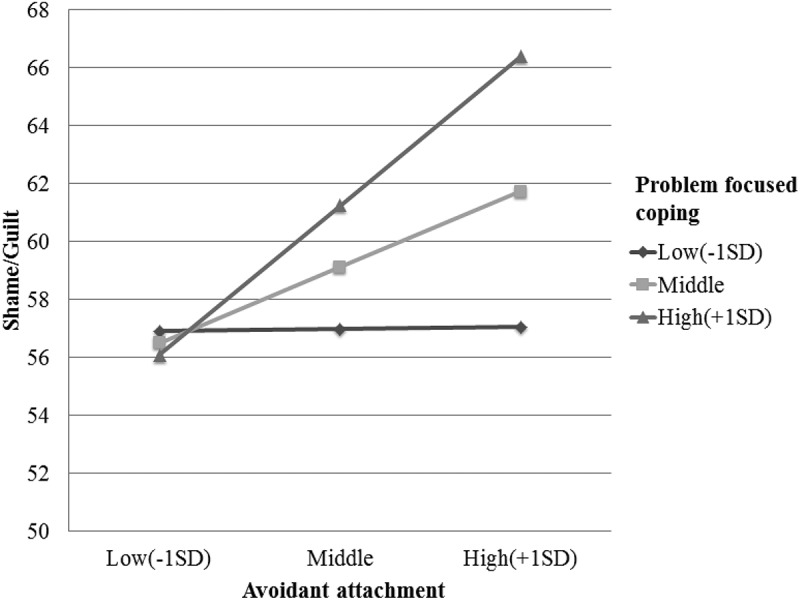
Moderation effect of problem focused coping in the relationship between avoidant attachment and shame/guilt.

Tables 3 and 4 summarize the results of our analysis of the moderating effect of coping strategy on the relationship between different styles of attachment and complicated grief and shame/guilt. The moderating effect of the problem-focused coping strategy was significant in the relationship between avoidant attachment and shame/guilt (*β* = 0.26, *p *< .05). To examine the pattern of the moderating effect, we calculated the dependent value according to three different levels of problem-coping strategy use (Mean, Mean + 1 *SD*, Mean – 1 *SD*) using regression equations (Table 5 and Figure 2). For the group with a high (+ 1 *SD*) problem-focused coping subscore, the results demonstrated a significant positive association between avoidant attachment and shame/guilt (*b* = 1.10, *p* < .01, 95% CI [0.40, 1.79]). For the groups with low (− 1 *SD*) and moderate problem-focused coping sub-scores, avoidant attachment was not significantly associated with shame/guilt.

**Table 3.** Moderation effect of coping strategy in the relationship between avoidant attachment, complicated grief, and shame/guilt.

	Complicated grief						Shame and guilt					
	B	*β*	*t*	*R*^2^	∆*R*^2^	*F*	B	*β*	*t*	*R*^2^	∆*R*^2^	*F*
Constant	52.45		32.98**	0.07	0.03	2.52	58.48		45.08**	0.06	0.06	3.43*
Avoidant attachment (X)	0.79	0.26	2.27*				0.61	0.24	2.14*			
Problem-focused coping (Z)	0.05	0.02	0.13				0.29	0.11	0.95			
XZ	−0.10	−0.17	−1.46	0.09			0.12	0.26	2.30*	0.12		
Constant	52.46		32.99**	0.07	0.02	2.41	58.47		45.18**	0.06	0.04	2.80*
Avoidant attachment (X)	0.76	0.25	2.21*				0.61	0.25	2.17*			
Emotion-focused coping (Z)	−0.07	−0.03	−0.22				0.31	0.13	1.14			
XZ	−0.07	−0.15	−1.33	0.09			0.08	0.20	1.76	0.10		
Constant	52.52		34.84**	0.16	0.03	5.75**	58.52		52.18**	0.30	0.01	10.98**
Avoidant attachment (X)	0.66	0.22	2.04*				0.37	0.15	1.55			
Avoidant coping (Z)	0.72	0.31	2.94**				0.95	0.50	5.22**			
XZ	−0.08	−0.16	−1.55	0.19			0.03	0.08	0.82	0.30		

*p < .05, **p < .01

**Table 4.** Moderation effect of coping strategy in the relationship between anxious attachment, complicated grief, and shame/guilt.

	Complicated grief						Shame and guilt					
	B	*β*	*t*	*R*^2^	∆*R*^2^	*F*	B	*β*	*t*	*R*^2^	∆*R*^2^	*F*
Constant	52.43		32.14**	0.02	0.02	0.88	58.49		46.28**	0.11	0.01	3.15*
Anxious attachment (X)	0.32	0.13	1.09				0.68	0.33	2.99**			
Problem-focused coping (Z)	−0.26	−0.08	−0.68				−0.10	−0.04	−0.33			
XZ	0.07	0.13	1.13	0.03			0.03	0.07	0.63	0.11		
Constant	52.44		32.26**	0.03	0.03	1.38	58.49		46.26**	0.11	0.03	4.02*
Anxious attachment (X)	0.35	0.14	1.20				0.67	0.33	2.93**			
Emotion-focused coping (Z)	−0.35	−0.12	−1.01				−0.06	−0.02	−0.20			
XZ	0.07	0.17	1.47	0.05			0.06	0.18	1.67	0.14		
Constant	52.50		33.91**	0.12	0.01	3.46*	58.52		52.91**	0.32	0.00	11.70**
Anxious attachment (X)	0.05	0.02	0.19				0.43	0.21	2.15*			
Avoidant coping (Z)	0.77	0.33	2.98**				0.89	0.47	4.87**			
XZ	−0.03	−0.08	−0.73	0.12			−0.01	−0.03	−0.26	0.32		

*p < .05, **p < .01

**Table 5.** Simple slope analysis about moderation effect of problem focused coping strategies in the relationship between avoidant attachment and shame/guilt.

		*b*	s.e.	*t*	LLCI(b)	ULCI(b)
Problem-focused coping	− 1 *SD*	0.02	0.38	0.04	−0.74	0.77
	M	0.56	0.28	2.00	0.00	1.11
	+ 1 *SD*	1.10	0.35	3.14	0.40	1.79

s.e. = Standard error, LLCI = Lower limit of confidence interval, ULCI = Upper limit of confidence interval.

## 4. Discussion

Despite increasing evidence that people who experience disaster-related bereavement are vulnerable to serious mental health problems, few studies have investigated the psychological factors that can affect the grief response to traumatic loss in disasters. Most previous studies of psychological factors affecting bereavement have been performed among parents who have lost a child or partner due to illness, such as cancer. Therefore, we aimed to examine the moderating role of coping strategies in the relationship between attachment style and grief outcomes in traumatic losses from human-caused disasters. The problem-focused coping strategy moderated the relationship between avoidant attachment and shame/guilt. In contrast to our hypothesis, a high level of problem-focused coping strengthened the positive association between avoidant attachment and negative self-conscious emotions.

We found anxious attachment to be positively correlated with shame/guilt and avoidant attachment to be correlated with the severity of complicated grief. Anxiously attached individuals have negative internal working models of the self (Bretherton & Munholland, 1999) and are concerned about their acceptability and worth. Traumatic loss can trigger and aggravate such negative self-views, which could contribute to negative self-conscious emotions, including shame and guilt. The association we found between avoidant attachment and complicated grief is consistent with the results of several previous studies about loss such as the death of a child (Meier, Carr, Currier, & Neimeyer, 2013; Wijngaards-de Meij et al., 2007b). Given the negative correlation we found between avoidant attachment and problem- and emotion-focused coping, we infer that individuals with avoidant attachment might have difficulty using adaptive/active coping strategies. Furthermore, the deactivating strategies, typical of avoidant attachment, might not be workable following a severe traumatic loss (Mikulincer, Dolev, & Shaver, 2004; Mikulincer, Shaver, & Pereg, 2003). Thus, inefficient coping related to avoidant attachment could lead to complicated grief. On the other hand, anxious attachment was not associated with complicated grief, which is inconsistent with previous results. Cultural issues or the specific situation of this accident might explain this inconsistent result. As mentioned above, these bereaved parents have existed as a group for a long time. Considering that social support might be a confounder between anxious attachment and grief response (van der Houwen et al., 2010), the collectivism of Eastern culture and the group’s collective action after the accident can influence on the present result. Further studies should be performed to clarify the relationships between social support/isolation, attachment, and grief among people with different cultural backgrounds.

In the relationship between attachment dimension and coping strategies, anxious attachment was positively associated with all types of coping strategies, and avoidant attachment was negatively associated with the two active coping strategies. The theoretical and empirical literature suggests that hyperactivating strategies are characteristic of anxious attachment, and deactivating strategies are common with avoidant attachment (Malik, Wells, & Wittkowski, 2015; Mikulincer et al., 2003). In a broad outline, our present results for the two types of active coping are in line with the theory that anxiously attached persons seem to hyperactively cope, and avoidantly attached individuals seem to deactively cope. However, the results for avoidant coping are somewhat inconsistent. It might be possible that some component that we categorized as avoidant coping in the Brief COPE (e.g. self-blame, venting) overlaps with the characteristics of anxious attachment’s secondary hyperactive strategies in extremely stressful situations. Further study with more elaborative assessment of coping strategies in a large sample would be necessary to clarify the relationship between avoidant coping and attachment dimension following extremely stressful events.

Contrary to our hypothesis, the positive association between avoidant attachment and negative self-conscious emotion was significant in the group with a high level of problem-focused coping. Based on attachment theory, avoidant-attached individuals use a positive self-view and a negative view of others as a form of self-defence (Bartholomew & Horowitz, 1991). They can be less susceptible to self-conscious emotions because of their preference for minimizing involvement and interdependence in relationships (Akbağ & Erden İmamoğlu, 2010; Mikulincer & Shaver, 2005; Muris et al., 2014). A relatively active coping strategy can threaten their defensive self-view, which they typically maintain by using deactivating or defensive strategies (Martins, Canavarro, & Moreira, 2016). Damage to their self-view triggered by problem-focused coping strategies might have provoked negative self-emotions, including shame/guilt. Furthermore, theoretically secure strategies of emotion regulation are three-fold and serial: acknowledgement and expression of distress, emotional support seeking, and engagement in instrumental problem solving (Waters, Rodrigues, & Ridgeway, 1998). The emotional-focused coping strategies, including acknowledging and displaying emotions and seeking emotional support, down-regulate distress so that problem-focused coping strategies, such as seeking instrumental support and solving problems, can proceed successfully (Lazarus & Folkman, 1984). Without active emotion-focused coping strategies to down-regulate emotional distress, problem-focused strategies might cause severe emotional distress in individuals with avoidant attachment. This finding has an important implication: among avoidantly attached individuals who have experienced traumatic loss, active problem-focused coping strategies could provoke overwhelming, negative self-conscious emotions unless sufficient emotional support is provided. They might be unable to integrate the traumatic loss into their life, making them more vulnerable to complicated grief. The present result suggests that close observation is necessary to help people with avoidant attachment in a traumatic loss even if they do not seek help for their painful experiences.

This study has several limitations. First, we included only a portion of all the bereaved parents due to the difficulty in obtaining consent because of social and political issues. As a result, our sample size was small. Most of the parents were angry and mistrusted the government and society. Furthermore, they had suffered from media attention and stigma. Therefore, some of them declined to participate in any more investigations, especially regarding their mental health. Selection bias should be considered because the study was limited to those who agreed to participate. Second, we used a cross-sectional study design, so we could not establish causal relationships between attachment style, coping strategies, and trauma-related emotions. Third, all variables, including attachment, were assessed using self-report methods. It is possible that many parents had distorted mental representations of their relationships with their dead children and their coping strategies. Therefore, the related results should be interpreted cautiously. Fourth, although the Brief COPE was categorized into three types of coping based on the theoretical and empirical literature, it is possible that the patterns for coping with an extremely stressful situation, such as losing a child, differ from the patterns following more usual stressful events. In addition, some coping strategies usually regarded as adaptive or active might not be workable or could even be harmful following a traumatic loss. Thus, categorization of the Brief COPE might not have been well fit to the participants’ situation. Although grouping the subscales using a principal component analysis would have been a better method, the sample was too small for such an analysis. Therefore, generalization of the present results should be done cautiously.

The strength of the present study is that the participants were a homogenous group who experienced traumatic loss in the same disaster. Our findings suggest that attachment style should be considered when helping individuals suffering from traumatic loss. In particular, individuals with avoidant attachment could have difficulties trying to adaptively cope because of overwhelming negative emotions caused by their extremely painful traumatic loss. It is possible that the interactive effects of attachment style and coping strategy influence longitudinal prognosis following a traumatic loss. Future studies should investigate the longitudinal effects of attachment style, coping strategy, and their interaction.

## References

[CIT0001] Aiken L. S., & West S. G. (1991). *Multiple regression: Testing and interpreting interactions*. Thousand Oaks, CA: Sage Publications.

[CIT0002] Akbağ M., & Erden İmamoğlu S. (2010). The prediction of gender and attachment styles on shame, guilt, and loneliness. *Kuram Ve Uygulamada Eğitim Bilimleri*, 10(2), 669–10.

[CIT0003] Barr P. (2004). Guilt- and shame-proneness and the grief of perinatal bereavement. *Psychology and Psychotherapy: Theory, Research and Practice*, 77(4), 493–510. doi:10.1348/1476083042555442 15588457

[CIT0004] Barr P. (2012). Negative self-conscious emotion and grief: An actor-partner analysis in couples bereaved by stillbirth or neonatal death. *Psychology and Psychotherapy: Theory, Research and Practice*, 85(3), 310–326. doi:10.1111/j.2044-8341.2011.02034.x 22903921

[CIT0005] Bartholomew K. (1990). Avoidance of intimacy: An attachment perspective. *Journal of Social and Personal Relationships*, 7(2), 147–178. doi:10.1177/0265407590072001

[CIT0006] Bartholomew K., & Horowitz L. M. (1991). Attachment styles among young adults: A test of a four-category model. *Journal of Personality and Social Psychology*, 61(2), 226–244. doi:10.1037/0022-3514.61.2.226 1920064

[CIT0007] Boelen P. A. (2016). Improving the understanding and treatment of complex grief: An important issue for psychotraumatology. *European Journal of Psychotraumatology*, 7(1), 32609. doi:10.3402/ejpt.v7.32609 27667723 PMC5035770

[CIT0008] Boelen P. A., de Keijser J., & Smid G. (2015). Cognitive-behavioral variables mediate the impact of violent loss on post-loss psychopathology. *Psychological Trauma: Theory, Research, Practice, and Policy*, 7(4), 382–390. doi:10.1037/tra0000018 26147521

[CIT0009] Boelen P. A., & Klugkist I. (2011). Cognitive behavioral variables mediate the associations of neuroticism and attachment insecurity with prolonged grief disorder severity. *Anxiety, Stress & Coping*, 24(3), 291–307. doi:10.1080/10615806.2010.527335 21069611

[CIT0010] Boelen P. A., van den Bout J., & van den Hout M. A. (2003). The role of negative interpretations of grief reactions in emotional problems after bereavement. *Journal of Behavior Therapy and Experimental Psychiatry*, 34(3–4), 225–238. doi:10.1016/j.jbtep.2003.08.001 14972670

[CIT0011] Boelen P. A., van den Bout J., & van den Hout M. A. (2006). Negative cognitions and avoidance in emotional problems after bereavement: A prospective study. *Behaviour Research and Therapy*, 44(11), 1657–1672. doi:10.1016/j.brat.2005.12.006 16457778

[CIT0012] Boelen P. A., van den Bout J., & van den hout M. A. (2010). A prospective examination of catastrophic misinterpretations and experiential avoidance in emotional distress following loss. *The Journal of Nervous and Mental Disease*, 198(4), 252–257. doi:10.1097/NMD.0b013e3181d619e4 20386253

[CIT0013] Bowlby J. (1980). *Attachment and loss*. New York, NY: Basic Books.

[CIT0014] Bretherton I., & Munholland K. A. (1999). Internal working models in attachment relationships: A construct revisited In Cassidy J. & Shaver P. R. (Eds.), *Handbook of attachment: Theory, research, and clinical applications* (pp. 89–111, Chapter xvii, 925 Pages). New York, NY: Guilford Press.

[CIT0015] Carver C. S. (1997). You want to measure coping but your protocol’s too long: Consider the Brief COPE. *International Journal of Behavioral Medicine*, 4(1), 92–100. doi:10.1207/s15327558ijbm0401_6 16250744

[CIT0016] Carver C. S., & Scheier M. F. (1994). Situational coping and coping dispositions in a stressful transaction. *Journal of Personality and Social Psychology*, 66(1), 184–195. 8126648 10.1037//0022-3514.66.1.184

[CIT0017] Carver C. S., Scheier M. F., & Weintraub J. K. (1989). Assessing coping strategies: A theoretically based approach. *Journal of Personality and Social Psychology*, 56(2), 267–283. doi:10.1037/0022-3514.56.2.267 2926629

[CIT0018] Coolidge F. L., Segal D. L., Hook J. N., & Stewart S. (2000). Personality disorders and coping among anxious older adults. *Journal of Anxiety Disorders*, 14(2), 157–172. 10864383 10.1016/s0887-6185(99)00046-8

[CIT0019] Cooper C., Katona C., & Livingston G. (2008). Validity and reliability of the brief COPE in carers of people with dementia: The LASER-AD Study. *The Journal of Nervous and Mental Disease*, 196(11), 838–843. doi:10.1097/NMD.0b013e31818b504c 19008735

[CIT0020] Endler N. S., & Parker J. D. A. (1994). Assessment of multidimensional coping: Task, emotion, and avoidance strategies. *Psychological Assessment*, 6(1), 50–60. doi:10.1037/1040-3590.6.1.50

[CIT0021] Field N. P., & Sundin E. C. (2001). Attachment style in adjustment to conjugal bereavement. *Journal of Social and Personal Relationships*, 18(3), 347–361. doi:10.1177/0265407501183003

[CIT0022] Folkman S., & Lazarus R. S. (1985). If it changes it must be a process: Study of emotion and coping during three stages of a college examination. *Journal of Personality and Social Psychology*, 48(1), 150–170. 2980281 10.1037//0022-3514.48.1.150

[CIT0023] Fraley R. C., & Bonanno G. A. (2004). Attachment and loss: A test of three competing models on the association between attachment-related avoidance and adaptation to bereavement. *Personality and Social Psychology Bulletin*, 30(7), 878–890. doi:10.1177/0146167204264289 15200694

[CIT0024] Fraley R. C., & Shaver P. R. (1997). Adult attachment and the suppression of unwanted thoughts. *Journal of Personality and Social Psychology*, 73(5), 1080–1091. doi:10.1037/0022-3514.73.5.1080 9364762

[CIT0025] George D., & Mallery P. (2010). *SPSS for Windows step by step: A simple guide and reference 17.0 update* (10th ed.). New Zealand, Auckland: Pearson Education.

[CIT0026] Harder D. W., & Zalma A. (1990). Two promising shame and guilt scales: A construct validity comparison. *Journal of Personality Assessment*, 55(3–4), 729–745. doi:10.1207/s15327752jpa5503&4_30 2280336

[CIT0027] Holahan C. J., & Moos R. H. (1987). Personal and contextual determinants of coping strategies. *Journal of Personality and Social Psychology*, 52(5), 946–955. doi:10.1037/0022-3514.52.5.946 3585703

[CIT0028] Huh H. J., Huh S., Lee S. H., & Chae J.-H. (2017). Unresolved bereavement and other mental health problems in parents of the sewol ferry accident after 18 months. *Psychiatry Investigation*, 14(3), 231–239. doi:10.4306/pi.2017.14.3.231 28539941 PMC5440425

[CIT0029] Jacobs S., Mazure C., & Prigerson H. (2000). Diagnostic criteria for traumatic grief. *Death Studies*, 24(3), 185–199. doi:10.1080/074811800200531 11010626

[CIT0030] Jerga A. M., Shaver P. R., & Wilkinson R. B. (2011). Attachment insecurities and identification of at-risk individuals following the death of a loved one. *Journal of Social and Personal Relationships*, 28(7), 891–914. doi:10.1177/0265407510397987

[CIT0031] Lazarus R. S. (1993). Coping theory and research: Past, present, and future. *Psychosomatic Medicine*, 55(3), 234–247. doi:10.1097/00006842-199305000-00002 8346332

[CIT0032] Lazarus R. S., & Folkman S. (1984). *Stress,appraisal, and coping*. New York, NY: Springer.

[CIT0033] Malik S., Wells A., & Wittkowski A. (2015). Emotion regulation as a mediator in the relationship between attachment and depressive symptomatology: A systematic review. *Journal of Affective Disorders*, 172, 428–444. doi:10.1016/j.jad.2014.10.007 25451448

[CIT0034] Martins T. C., Canavarro M. C., & Moreira H. (2016). Adult attachment and dyadic adjustment: The mediating role of shame. *The Journal of Psychology: Interdisciplinary and Applied*, 150(5), 560–575. doi:10.1080/00223980.2015.1114461 26759960

[CIT0035] Meier A. M., Carr D. R., Currier J. M., & Neimeyer R. A. (2013). Attachment anxiety and avoidance in coping with bereavement: Two studies. *Journal of Social and Clinical Psychology*, 32(3), 315–334. doi:10.1521/jscp.2013.32.3.315

[CIT0036] Mikulincer M., Birnbaum G., Woddis D., & Nachmias O. (2000). Stress and accessibility of proximity-related thoughts: Exploring the normative and intraindividual components of attachment theory. *Journal of Personality and Social Psychology*, 78(3), 509–523. doi:10.1037/0022-3514.78.3.509 10743877

[CIT0037] Mikulincer M., Dolev T., & Shaver P. R. (2004). Attachment-related strategies during thought suppression: ironic rebounds and vulnerable self-representations. *Journal of Personality and Social Psychology: Personality Processes and Individual Differences*, 87(6), 940–956. doi:10.1037/0022-3514.87.6.940 15598116

[CIT0038] Mikulincer M., Gillath O., & Shaver P. R. (2002). Activation of the attachment system in adulthood: Threat-related primes increase the accessibility of mental representations of attachment figures. *Journal of Personality and Social Psychology*, 83(4), 881–895. doi:10.1037/0022-3514.83.4.881 12374442

[CIT0039] Mikulincer M., & Shaver P. R. (2005). Attachment theory and emotions in close relationships: Exploring the attachment-related dynamics of emotional reactions to relational events. *Personal Relationships*, 12(2), 149–168. doi:10.1111/j.1350-4126.2005.00108.x

[CIT0040] Mikulincer M., Shaver P. R., & Pereg D. (2003). Attachment theory and affect regulation: The dynamics, development, and cognitive consequences of attachment-related strategies. *Motivation and Emotion*, 27(2), 77–102. doi:10.1023/A:1024515519160

[CIT0041] Muris P., Meesters C., Cima M., Verhagen M., Brochard N., Sanders A., … Meesters V. (2014). Bound to feel bad about oneself: Relations between attachment and the self-conscious emotions of guilt and shame in children and adolescents. *Journal of Child and Family Studies*, 23(7), 1278–1288. doi:10.1007/s10826-013-9817-z

[CIT0042] Neria Y., Gross R., Litz B., Maguen S., Insel B., Seirmarco G., … Marshall R. D. (2007). Prevalence and psychological correlates of complicated grief among bereaved adults 2.5-3.5 years after September 11th attacks. *Journal of Traumatic Stress*, 20(3), 251–262. doi:10.1002/jts.20223 17597124

[CIT0043] Prigerson H. G., Bierhals A. J., Kasl S. V., Reynolds C. F. 3rd, Shear M. K., Day N., … Jacobs S. (1997). Traumatic grief as a risk factor for mental and physical morbidity. *American Journal of Psychiatry*, 154(5), 616–623. doi:10.1176/ajp.154.5.616 9137115

[CIT0044] Prigerson H. G., Maciejewski P. K., Reynolds C. F. 3rd, Bierhals A. J., Newsom J. T., Fasiczka A., … Miller M. (1995). Inventory of Complicated Grief: A scale to measure maladaptive symptoms of loss. *Psychiatry Research*, 59(1–2), 65–79. 8771222 10.1016/0165-1781(95)02757-2

[CIT0045] Schnider K. R., Elhai J. D., & Gray M. J. (2007). Coping style use predicts posttraumatic stress and complicated grief symptom severity among college students reporting a traumatic loss. *Journal of Counseling Psychology*, 54(3), 344–350. doi:10.1037/0022-0167.54.3.344

[CIT0046] Suar D., Alat P., & Das S. S. (2017). Attachment styles predicting posttsunami trauma: The mediating effects of coping. *Psychological Studies*, 62, 160–167. doi:10.1007/s12646-017-0399-5

[CIT0047] Tabachnick B. G., & Fidell L. S. (2007). *Using multivariate statistics* (5th ed.). Boston, MA: Allyn & Bacon/Pearson Education.

[CIT0048] van der Houwen K., Stroebe M., Schut H., Stroebe W., & van den Bout J. (2010). Mediating processes in bereavement: The role of rumination, threatening grief interpretations, and deliberate grief avoidance. *Social Science & Medicine*, 71(9), 1669–1676. doi:10.1016/j.socscimed.2010.06.047 20832924

[CIT0049] Waters H. S., Rodrigues L. M., & Ridgeway D. (1998). Cognitive underpinnings of narrative attachment assessment. *Journal of Experimental Child Psychology*, 71(3), 211–234. doi:10.1006/jecp.1998.2473 9878106

[CIT0050] Wayment H. A., & Vierthaler J. (2002). Attachment style and bereavement reactions. *Journal of Loss and Trauma*, 7(2), 129–149. doi:10.1080/153250202753472291

[CIT0051] Wei M., Russell D. W., Mallinckrodt B., & Vogel D. L. (2007). The Experiences in Close Relationship Scale (ECR)-short form: Reliability, validity, and factor structure. *Journal of Personality Assessment*, 88(2), 187–204. doi:10.1080/00223890701268041 17437384

[CIT0052] Wijngaards-de Meij L., Stroebe M., Schut H., Stroebe W., van den Bout J., van der Heijden P., & Dijkstra I. (2007a). Neuroticism and attachment insecurity as predictors of bereavement outcome. *Journal of Research in Personality*, 41(2), 498–505. doi:10.1016/j.jrp.2006.06.001

[CIT0053] Wijngaards-de Meij L., Stroebe M., Schut H., Stroebe W., van den Bout J., van der Heijden P. G. M., & Dijkstra I. (2007b). Patterns of attachment and parents’ adjustment to the death of their child. *Personality and Social Psychology Bulletin*, 33(4), 537–548. doi:10.1177/0146167206297400 17363759

